# Evaluation of Er,Cr:YSGG Laser Effect on Microshear Bond Strength of a Self-Adhesive Flowable Composite in the Dentin of Permanent Molar: An In Vitro Study

**DOI:** 10.1155/2016/4856285

**Published:** 2016-07-14

**Authors:** Masoumeh Moslemi, Faezeh Fotouhi Ardakani, Fatemeh Javadi, Zahra Khalili Sadrabad, Zahra Shadkar, Mohammad Saeid Shadkar

**Affiliations:** ^1^Pediatric Department, Faculty of Dentistry, Shahid Beheshti University of Medical Sciences, Tehran, Iran; ^2^Pediatric Department, Faculty of Dentistry, Shahid Sadoughi University of Medical Sciences, Yazd 8914815667, Iran; ^3^Pediatric Department, Faculty of Dentistry, Shahed University of Medical Sciences, Tehran, Iran; ^4^Private Practice, Tehran, Iran; ^5^Faculty of Economics, University of Tehran, Tehran, Iran

## Abstract

*Aim and Background*. Recently, new restorative materials such as self-adhesive flowable composites, because of their simple use and no need to bonding and etching, are considered important, particularly in pediatric dentistry. The aim of this study is to evaluate the effect of Er,Cr:YSGG laser on microshear bond strength of self-adhesive flowable composite on permanent teeth dentin in vitro.* Material and Methods*. In this experimental study, 40 dentin sections were prepared from healthy third molars and divided into two groups according to their surface preparation by Er,Cr:YSGG laser or without laser, only with silicon carbide paper. In each group, two groups of 10 teeth were treated with self-adhesive flowable composite (Dyad) and conventional flowable composite (acid etch and bonding). Samples were stored in normal saline and after 48 hours their bond strength was measured. The failure mode of samples was observed on stereomicroscope. In order to analyse the results, the one way ANOVA and Tukey's test for multiple comparisons were used.* Result*. The maximum bond strength was related to conventional flowable composite with laser preparation group (24/21 Mpa). The lowest one was seen in Dyad composite without laser emitting (9/89 Mpa). The statistical difference between this two groups was significant (*P* value = 0/0038). The microshear bond strength differences between Dyad composite groups with laser preparation (mean = 16/427 ± 1/79) and without laser preparation (mean = 12/85 ± 1/90) were statistically significant too (*P* value = 0/01).* Conclusion*. Self-adhesive flowable composite has lower microshear bond strength than conventional flowable composite. Moreover, the laser irradiation as a surface treatment can improve this bond strength.

## 1. Introduction

The demand for cosmetic services and maintenance of the tooth structure leads to further development of adhesive materials which act through bonding to the enamel and dentin. Flowable resin composite has become popular due to its less viscosity and hence less need for tooth preparation in superfacial restorations and preventive resin restorations. The new generation of these composites, the so-called self-adhesive flowable composites, can be bonded to tooth structures without using adhesive systems according to its manufactures' claim. The aforementioned feature of these composites is due to acidic monomer in its composition. Another feature includes GPDM monomer, that is, functional phosphate group, which bonds chemically to carbon ions in tooth surface, and etches dentin and enamel. On the other hand, there are two functional methacrylate groups which polymerized by other methacrylate monomers which result in adhesive mechanical strength improvement. Moreover, its tritium fluoride particles make its opacity up to 320% for easy diagnosis in radiography. The bond strength of this composite is similar to other self-etch adhesives [1].

There are some problems associated with using these adhesive systems such as long treatment time and high technical sensitivity. The aforementioned self-adhesive flowable resins are also helpful in treating uncooperative patients [2]. There are some factors that affect bond strength of resin to tooth structure such as the type of adhesive system, restorative materials, and cavity preparation. Recent research works emphasize on the necessity of better tooth preparation to enhance penetration of resin to tooth structure. Erbium laser has a number of advantages, for example, lack of the creation of the vibration, sound, and stress and reducing the need of local anesthesia. Waterlase and Biolase possess Er,Cr:YSGG laser energy with a wavelength of 2780 nm with a capability of air-water spray.

Patent atomizing waterlase spray simultaneously stimulates the water molecules in spray and also in the target tissue, so this can cause biological ruptures in microscale in the tooth structure as well as on the bone and the soft tissue [3].

The energy of waterlase Er,Cr:YSGG laser is superior to that of Er:YAG laser. This is because the Erbium laser quickly vaporizes water in the dentinal tubules and enamel prisms of the hard tissues. This fact leads to less energy absorption by hydroxyapatite crystals and enamel, which will in turn cause a decrease in laser cutting speed and hence an ultimate effect of tissue damage [4].

The waterlase laser handpiece never touches the tooth. In this way, by eliminating the vibrations, it protects the tooth from microscopic cracks and also prevents making groove on the adjacent tooth surfaces [4]. The laser effects on dental tissues consist of thermomechanical wear and evaporation of water content. This causes expansion and disposal of organic and inorganic tissue contents and ultimately a surface with open dentinal tubules without smear layer [5, 6]. The bond strength to tooth surfaces prepared with laser is often confusing and accompanied with contradictive results. Some studies show that the bond strength to tooth surfaces which was prepared by low-power Er:YAG and Er,Cr:YSGG laser is less than the bond strength to surfaces with acid-induced conditioning [1, 5, 7].

In some other studies the laser efficiency in conditioning the tooth surface could become comparable to that with acid etch by changing some variables that belong to the laser equipment, for example, its distance from the tooth surface and the output power [6].

Ozel Bektas et al. concluded in his study that the highest shear bond strength was dedicated to OVF (optibond + vertise flow) group [8].

In 2013 Yazici et al. showed that the laser irradiation, as a tool for conditioning the dentin surfaces, increases the bond strength of self-adhesive composites [9]. This is whereas some other studies, for example, Wajdowicz et al. study, suggest that the laser irradiation is not effective in increasing the bond strength [10]. Therefore the primary objective of the present study was to compare the bond strength of a self-adhesive flowable composite resin to dentin surface followed by acid etch and laser conditioning.

## 2. Methods and Materials

The ethical approval for this experimental in vitro study was obtained from the ethics committee of Shahid Beheshti University of Medical Sciences, Tehran, Iran. In this study, 40 healthy, caries-free human third molar teeth were collected during 2 months. Prior to the commencement of our study the teeth were immersed in chloramine solution for a period of 1 week to be disinfected and then were stored in a normal saline solution.

The teeth roots were sectioned at a 2 mm distance above the CEJ by a cutting device (Hamco machine, IW, Rochester, NY, Patent pending). The occlusal enamel surfaces were removed using a diamond fissured bur (Kavo, USA) at high speed with air/water spray. In the next step they were grinded by silicon sandpaper (Mardor, Germany), once by 400 grits and another time by 800 grits, in order to attain the most superfacial dentin layer with the presence of the enamel adjacent to the aforementioned dentin. In this way 40 dentin sections were prepared which were divided to 2 groups of 20 samples each.

*Group 1*. The samples were exposed to Er,Cr:YSGG laser irradiation after preparation by silicon carbide sandpaper. A 2780 nm wavelength waterlase laser (Biolase Technology) (San Clement, CA, USA) with 140 ms pulse time, a 20 Hz repetition rate (i.e., frequency), and variable output power in the range of 0–6 watts was used.

In this study, the type of laser was G6 with a length and a diameter of 6 and 0–6 mm, respectively. Moreover the laser output power was set to be 2 watts, with the diameter laser beam of 1 mm with 50% water and 50% air. The power density of 254 w/cm^2^ was employed.

*Group 2*. The samples were exposed to Er,Cr:YSGG laser irradiation without preparation by silicon carbide.

Each group was further divided into two subgroups and prepared as follows:

- self-adhesive flowable composites (Dyad Flow/Kerr) with no acid etching step and bonding,
- conventional flowable composite (3M/Dental Product, USA) with acid etch conditioning and bonding (single-bond, 3M ESPE Dental Product, USA).

The composite placement on the tooth surfaces was performed by Tygon tubes (R 3603, Norton Performance Plastic Co, Cleveland) with a diameter and height of 0.7 and 1 mm, respectively. Firstly the samples were etched by brushes weltered in 37% phosphoric acid liquid for 15 seconds. In the next step the samples were rinsed with the water spray for 15 seconds and then dried by the air-spray for 10 seconds (in compliance with the company's instructions).

Secondly two layers of single bond were used once rinsing and drying completed. This was followed by light curing for 10 seconds with a visible light curing unit (XL 3000, 3M Dental Products, USA).

Finally, the Tygon tube cylinders were placed on tooth surfaces with the height of 1 mm and light cured for 40 seconds. The placement of the self-adhesive composite was accomplished according to manufacturer's instructions. The samples were stored in the distilled water at a temperature of 37°C for 1 hour prior to removing the Tygon tubes by a scalpel. As the last step the samples were restored in 37°C water for 24 hours.

The microshear strength of samples was measured by microshear machine with a crosshead speed of 0.5 mm/min. In the next step, the bond strength was calculated through the following equation:

(1) microshear  bond  strength  of  composite=bonding  force  Newtoncrosscut  section  of  composite  (mm).

The type of fracture that occurred in each sample was examined under a stereomicroscope (Olympus Optical Co., Ltd., model SZXLL b 2000 Japan) at a magnification of 12.5x. Moreover the one way ANOVA and Tukey's multiple comparison were used. The type 1 error was assumed in this study.

## 3. Results

The statistical metrics of the microshear strength of dentin samples are outlined in Table 1 when the one way ANOVA is taken advantage of.

**Figure 1:**
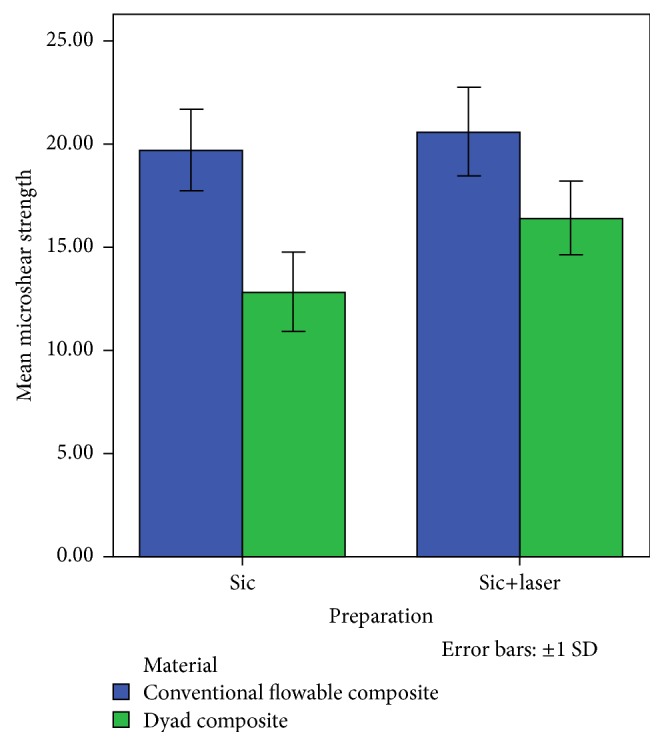
The mean microshear bond strength values in different groups.

The highest microshear strength value was observed in conventional flowable composite group which was prepared by silicon carbide and laser irradiation. On the other hand, the lowest microshear bond strength value was observed in Dyad composite group which was prepared by silicon carbide (Figure 1). It is observed that the difference between the aforesaid values was statistically significant (*P* value = 0.003).

Taking into account the significant results of the ANOVA test, a one to one comparison between two groups was performed throughout the Tukey's test (see Table 2 for the results).

In accordance with the Tukey's HSD analysis, there was a significant difference between laser and silicon carbide preparation in Dyad composite group (*P* value = 0.01).

The failure mode of samples based upon stereomicroscope observations showed. The adhesive failure was the most frequent among samples particularly in group 4, that is, Dyad composite without laser irradiation which experienced the adhesive failure in 100% of samples.

## 4. Discussion

To the best knowledge of the authors of this paper, very few studies have been conducted so far on the microshear bond strength of the new generation self-adhesive dentin composites in the permanent teeth. This fact was the primary motivator of our work in this paper on the microshear bond strength of the most recent self-adhesive composite, that is, Dyad composite.

The results showed that the highest microshear bond strength belonged to the flowable conventional composite group under Er,Cr:YSGG laser surface preparation. The lowest microshear bond strength was observed in self-adhesive Dyad composite category without laser irradiation. The difference between the aforementioned categories was statistically significant (*P* value = 0.0038). Recently the use of laser to increase adhesion and resin bond to tooth structure is recommended. The laser is also suggested for the etching of tooth surface as an alternative way to acid etching. The effectiveness and the efficiency of this method are still being worked on. While some researchers have shown laser ability for etching and dentin surface preparation [9, 11, 12], some others have denied its ability [10].

In the present study the microshear bond strength of Dyad self-adhesive composite with and without laser irradiation had a significant difference (*P* value = 0.01). This observation can be explained and justified as follows.

The preparation of tooth surface by rotary instrument produces an amorphous organic and inorganic layer of debris depositing on the surface. The aforesaid smear layer is resistant to mechanical ablation and can only be removed through chemical solutions. This smear layer prevents resin monomers penetration into the dentin structure in order to achieve sufficient bond to dentin. The smear layer has to be removed before using the adhesive material. The blockage of dentinal tubules with smear layer, as a result of preparation by silicon carbide paper, could cause incomplete penetration of Dyad self-adhesive composites. This will eventually lead to lower microbond strength values in comparison with conventional composites that have a classical etching and rinsing system. The dihydrogen transferase glycerol methacrylate is a self-etching/self-adhering acidic monomer in Dyad flowable composite and has been used in single bond. The above-said acidic monomer is not acidic enough to remove smear layer. The PH level of that monomer increases from 1.9 to 6.5–7 during polymerization. One of the advantages of this kind of monomer is fluoride releasing due to presence of ytterbium fluoride in the composition. To ensure sufficient contact of the composite to the tooth structure, it is required to use medium pressure for 15–20 seconds when brushing the first layer of composite to the tooth walls. Different studies show that the bond strength of Dyad flow composite to surfaces prepared by laser is higher than the ones prepared by silicon carbide paper. This is due to three features of the tooth surface that are as a result of laser irradiation: (1) roughness of the surface, (2) open dentinal tubules, and (3) the lack of smear layer on the surface [13]. As it was also justified in Gurgan et al. study [14], our study indicates a nonsignificant difference on the bond strength of the conventional flowable composite, when comparing laser and silicon carbide groups. Conventional flowable composite was used according to the manufacturer's instructions, based on which acid etching was used after the laser irradiation. Previous studies showed that laser irradiation does not eliminate the need for acid etching [15–17]. Nevertheless, there is no surprise that corresponding bond strengths indicate similar values. The Er,Cr:YSGG laser causes microexpansions in the mineral tooth structures according to Chen study [18–20]. Another study has concluded that morphological changes induced by Er,Cr:YSGG laser adversely affect the performance of bond and adhesive [19, 21]. One other study concluded that the bond strength of surfaces irradiated by Er:YAG laser was lower than the ones irradiated by silicon carbide paper or diamond bur. This is due to SEM observations that show loss of fibrillar collagen in the subsurface dentinal area. This caused the elimination of* free spaces* among fibrils and prevention of hybridization [22].

According to the observations the adhesive failure was the most frequent type of fracture and within which group number 4 was the most prevalent. 100 percent of samples in group 4 were observed suffering adhesive failure. These results were in-line with the ones drawn by Yazici et al. [9].

One of the notable points in this study was the relatively high percentage of cohesive failure in group 1, that is, conventional flowable composite under laser irradiation. 40% of the samples experience this type of fracture which is attributed to strong bonding due to surface preparation by acid etching with laser irradiation and use of single bond or high substrate degradation.

The comparison between this study and previous works in this field demonstrated that different effects of laser irradiation on the tooth surface were due to the use of laser parameters such as output power and the distance between the laser tube and the tooth surface. Such different and contradictive results mentioned above were also experienced when comparing the results of articles due to various underlying conditions such as wide range of laser parameters, the type of tooth preparation, and adhesive restorative material. It is crucial to choose appropriate laser parameters that prevent undesirable changes on the tooth structure and also negative effects on the bond strength of the restorative materials to tooth surface [23].

## 5. Conclusion

The bond strength of self-adhesive flowable composite depends upon the type of tooth surface preparation. The laser conditioning of the tooth surfaces increased the bond strength of Dyad flowable composite to the tooth dentin. The highest bond strength belonged to flowable composite when prepared by Er,Cr:YSGG laser irradiation.

## Figures and Tables

**Table 1:** The statistical metrics of the microshear bond strength of dentin samples.

Whole number	Standard deviation	Mean bond strength	Maximum bond values (Mpa)	Minimum bond values (Mpa)	Type of material	Surface preparation
10	±2/16	20.62	24.21	17/7	^*∗*^(1)	Laser + silicon carbide paper
10	±1/79	16.427	18.75	15.1	^*∗*^(2)	
20	±2/56	18.749	24.21	15.1	Whole number	

10	±1/99	19.724	22.32	16.8	^*∗*^(3)	Silicon carbide paper
10	±1/90	12.85	15.88	9.89	^*∗*^(4)	
20	±3/9	17.59	22.32	9.89	Whole number	

^*∗*^3, ^*∗*^1: conventional flowable composite

^*∗*^4, ^*∗*^2: Dyad self-adhesive composite.

**Table 2:** *One to one* comparison between mean microshear bond strength between different dentinal groups according to Tukey's test.

	Groups	Mean difference	Standard error	Sig.
1	2	4.19300^*∗*^	.88051	.000^*∗*^
	3	.89600	.88051	.740
	4	7.77000^*∗*^	.88051	.000^*∗*^

2	1	−4.19300^*∗*^	.88051	.000^*∗*^
	3	−3.29700^*∗*^	.88051	.003^*∗*^
	4	3.57700^*∗*^	.88051	.001^*∗*^

3	1	−.89600	.88051	.740
	2	3.29700^*∗*^	.88051	.003^*∗*^
	4	6.87400^*∗*^	.88051	.000^*∗*^

4	1	−7.77000^*∗*^	.88051	.000^*∗*^
	2	−3.57700^*∗*^	.88051	.001^*∗*^
	3	−6.87400^*∗*^	.88051	.000^*∗*^
